# Four circadian rhythm-related genes predict incidence and prognosis in hepatocellular carcinoma

**DOI:** 10.3389/fonc.2022.937403

**Published:** 2022-11-10

**Authors:** Zhenyu Wu, Hao Hu, Qiang Zhang, Tengfei Wang, Huixing Li, Yugang Qin, Xiangnan Ai, Wen Yi, Xiaojun Wei, Wei Gao, Caiguo Ouyang

**Affiliations:** Department of Hepatobiliary Surgery, Aerospace Center Hospital, Beijing, China

**Keywords:** liver cancer, Circadian clock, overall survival, gene signature, immune

## Abstract

Circadian dysregulation can be involved in the development of malignant tumors, though its relationship with the progression of hepatocellular carcinoma is not yet fully understood. We identified genes related to circadian rhythms from the Cancer Genome Atlas (TCGA), measured gene expression, and conducted genomic difference analysis to construct a circadian rhythm-related signature. The resulting prognosis model proved to be an effective biomarker, as demonstrated by Kaplan-Meier survival analysis for both the training (n = 370, P = 2.687e-10) and external validation cohorts (n = 230, P = 1.45e-02). Further, we found that patients considered ‘high risk’, with an associated poor prognosis, displayed elevated levels of immune checkpoint genes and immune filtration. We also conducted functional enrichment, which indicated that the risk model showed a significant positive correlation with certain malignant phenotypes, including G2M checkpoint, MYC targets, and the MTORC1 signaling pathway. In summary, we identified a novel circadian rhythm-related signature allowing assessment of prognosis for hepatocellular carcinoma patients, and further can be used to predict immune infiltration sensitivity.

## Introduction

Hepatocellular carcinoma, the most common form of liver cancer, is one of the most common causes of cancer-related deaths worldwide, and is associated with substantial costs (1). Potential treatments at early stages include liver resection, ablation, and transplantation, though most HCC patients are diagnosed at later stages, limiting treatment options (2). Treatments for advanced-stage HCC are primarily systemic therapy approaches, including immune checkpoint inhibitors and targeted agents (3). Despite these available treatments, HCC patients generally experience recurrence and chemoresistance, resulting in overall poor prognosis. Empowered by innovations in multi-omics profiling, contemporary studies have provided prognostic candidates with clinical applications. In order to further the field, it is critical to identify strong molecular biomarkers that correlate with outcomes for HCC patients (4).

The molecular circadian clock is an evolutionarily conserved system that coordinates internal time with the external environment. The circadian clock is integral in homeostasis and regulation, and manages many biological activities and behaviors (5, 6). Within the hypothalamus, the suprachiasmatic nucleus (SCN) has a central clock that regulates daily rhythms through neural and humoral coordination of additional clocks found in peripheral tissues and organs, and these clocks control physiological functions including sleep/wake cycles and hormone secretion. The World Health Organization lists circadian clock disruption as a putative carcinogen, as evidenced by population and laboratory-based findings (7, 8). Thus, research into the relationship between the expression of circadian-related genes and tumor development has been a focus. A previous study demonstrated that circadian-related genes are correlated with the incidence and progression of NSCLC (9). Further research is required to fully explore the relationship between circadian genes and the prognosis for LIHC patients (10).

In the current study, we investigate the predictive role of circadian genes in LIHC patients using The Cancer Genome Atlas (TCGA) data from the NCI Genomic Data Commons, including clinical characteristics and the SNV, CNV, methylation, and mRNA expression profiles of tumor and tumor-adjacent normal tissues. We use these data to establish a prognostic multigene signature with differentially expressed circadian clock genes and validate this signature with the ICGC dataset. Essential molecular mechanisms are explored by gene set enrichment analysis (GSEA) using Kyoto Encyclopedia of Genes and Genomes (KEGG) pathways (10).

## Materials and methods

### Data collection and processing

Multi-omics data profiles for the 14 circadian genes (10–13) from 374 HCC patients together with their clinical data were obtained from TCGA LIHC datasets (https://cancergenome.nih.gov/). Heatmap was completed to visualize expression levels of the 14 circadian genes in HCC and of the normal tissues in the TCGA LIHC cohort. RNA-seq data of 232 HCC cases were also obtained from the ICGC cohort (https://dcc.icgc.org/projects/LIRI-JP). Clinical data for the TCGA and ICGC cohorts are displayed in **Table 1**. Transcriptome RNA sequencing data in the count and FPKM format and corresponding clinical data were acquired from TCGA (https://portal.gdc.cancer.gov/) and used as the training dataset. Genes with average expression < 0.5 were omitted from the current study. The EdgeR package in R was applied for genomic difference detection with |logFC| > 1 and adjusted P < 0.05 filtering. Volcano plot was created with the ggplot2 package to visualize the difference analysis.

**Table 1:** Demographical and clinical characteristics.

	level	ICGC_LIRI	TCGA_LIHC	p
n		228	370	
gender	female	59 (25.9)	121 (32.7)	0.094
	male	169 (74.1)	249 (67.3)	
age_at_index.mean.SD		67.18 (10.17)	59.42 (13.53)	<0.001
stage	1	35 (15.4)	170 (49.1)	<0.001
	2	104 (45.6)	86 (24.9)	
	3	70 (30.7)	85 (24.6)	
	4	19 (8.3)	5 (1.4)	
time.mean.SD.		814.74 (417.21)	802.91 (726.97)	0.823
status	0	186 (81.6)	240 (64.9)	<0.001
	1	42 (18.4)	130 (35.1)	

### Survival analysis

The survival package in R was used for Kaplan-Meier survival analysis with log-rank test, and for univariate and multivariate Cox regression. The glmnet R package was used for Lasso, and 10-fold cross-validation was implemented. Calibration plots were created with the rms package. The survivalROC package was used to create the time-dependent ROC curves.

Univariate Cox regression test was implemented to evaluate the correlation of circadian-related genes with clinical outcomes for HCC patients. Circadian-related genes with P<0.05 were included in further analysis with the Univariate Cox regression test with the least absolute shrinkage and selection operator (LASSO) algorithm with the “glmnet” package in R. A 4 gene predictive signature was screened using the minimum criteria. Risk score for each patient was computed using the normalized expression level of each gene as well as its coefficients. The formula was the following: coefficient (gene i) derived from the Cox proportional hazards regression analysis.

The TCGA LIHC cohort was split into two sub-groups on the basis of the median value of the risk score.

### GSEA and GSVA

Gene ontology (GO) was employed to investigate potential functions based on the differential gene profiles of the two groups (|log2FC| ≥ 1, FDR <0.05). The hallmark gene sets v. 7.4 were obtained from MSigDB and used as the reference dataset. GSVA was carried out with the GSVA package of R, and parameters were the following: min. size = 10, max. size =500, verbose = Ture, and parallel. size = 1. Difference detection was conducted with Limma, with the filtering threshold |logFC| > 0.1 and adjusted P < 0.05. GSEA was conducted with GSEA software (version 4.1.0), with the number of permutations as 1000 (14). The gene sets with nominal P < 0.05 and FDR q <0.05 were considered statistically significant. An interaction network of 14 circadian-related genes was recapitulated with STRING (http://string-db.org).

### Chemotherapeutic effectiveness analyses

The oncoPredict R package was used to assess the chemotherapeutic sensitivity of LIHC patients in the TCGA-LIHC cohort (15). Transcriptome data and IC50 values of 20 different cell lines were obtained from the GDSC database (https://www.cancerrxgene.org/) in order to corroborate the predictive value of CRRS for response to chemotherapy. Microarray RNA expression data retrieved from the GDSC dataset was normalized using Robust Multi-Array Average (RMA).

### Statistical analysis

R software (version 4.1.0) was used for statistical analysis. The Wilcoxon Signed-rank test was used for continuous variables from the bioinformatical analyses. Violin diagrams and boxplots were both created with the ggplot2 package. Chi-square test was employed for categorical variables, and results were visualized using the ggplot2 package.

## Results

### Expression profile of circadian clock genes and clinical features in TCGA LIHC cohort

**Figure 1:**
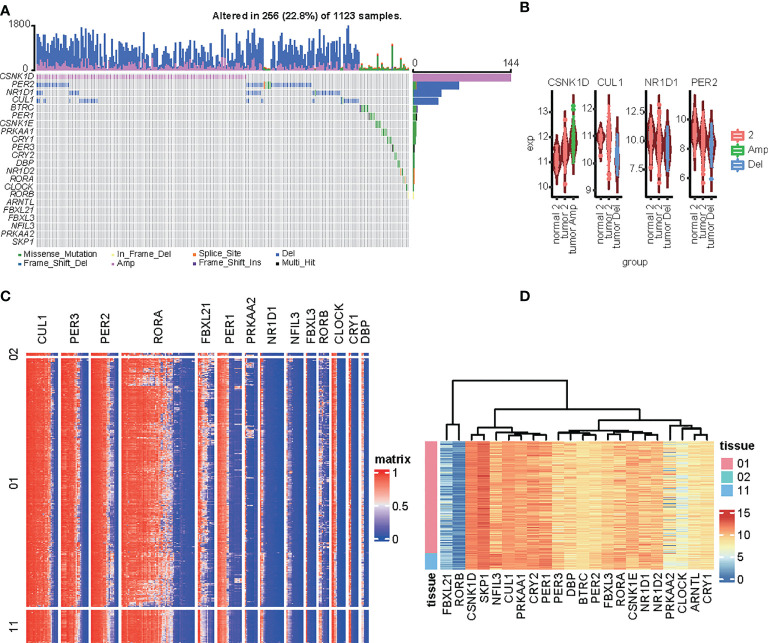
Genomic alterations to circadian-related genes in HCC tissues. **(A)** Oncoprint of 14 circadian-related genes in the TCGA LIH dataset generated by maftools. **(B)** Comparison of the expression of circadian-related genes among amplification, deletion, or normal in the LIHC dataset. **(C, D)** Heatmap of the circadian-related gene methylation types **(C)** and expression **(D)** in the LIHC dataset.

We included circadian clock genes (CCGs), including both core clock genes and clock control genes that were found in previous studies in our analysis (11, 16). SNVs and CNVs related to CCGs were investigated. Samples with mutated or CNVs CSNK1D, PER2, NR1D1, CUL1, and BTRC accounted for 1% of all samples from the TCGA dataset (**Figure 1A**). Additionally, the CNV profile of core CCGs could explain the abnormal expression of the CCGs. Expression of CSNK1D was increased in tumor CNV amplification samples relative to nonapplication samples and normal tissues, which corresponds with the CNV profile of the CCGs. The expression levels of CUL1, NR1D1, and PER2 were lower in tumor deletion samples than in other tumor and normal tissue samples (**Figure 1B**). Methylation and expression profiles of the CCGs between normal liver tissue and hepatocellular carcinoma samples is shown in the heatmap (**Figures 1C, D**). High methylation was observed for CLU1, PER3, PER3, RORA, and PER1, but was not associated with RNA expression; these genes showed high expression in the LIHC datasets. Low expression was observed for FBXL21 and RORB, but the methylation levels were not elevated. For FBXL21, only the partial methylation site was elevated. No significant relationship was observed between the methylation and gene expression of CCGs in LIHC.

### Construction of the circadian gene based signature in the TCGA LIHC cohort

**Figure 2:**
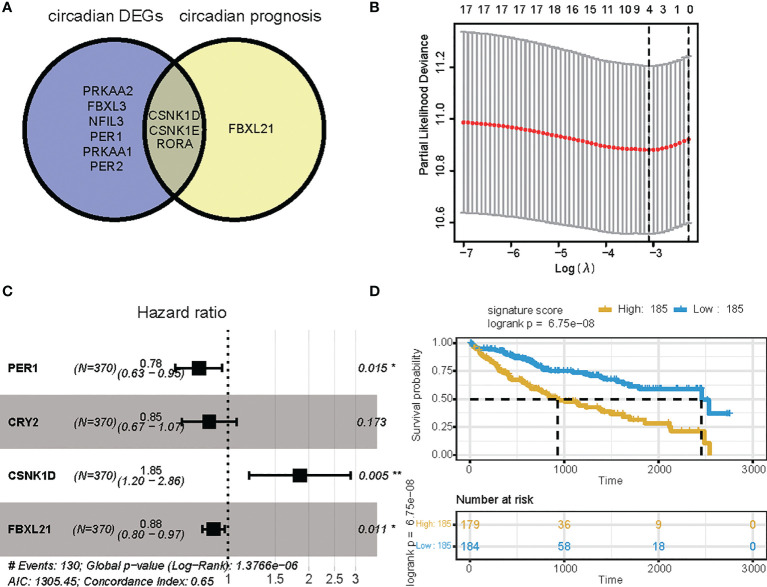
Survival analysis based on the risk score model. Survival analysis of the LIHC cohort in the TCGA. **(A)** Venn diagram of DEGs and prognostic genes that correlate with OS in tumor and tumor-adjacent normal tissue. **(B)** Lasso regression identified 4 genes correlated with prognosis. **(C)** Forest plots of the four genes that overlap between DEGs or prognostic genes that relate to OS based on univariate Cox regression analysis. **(D)** Kaplan Meier curves for the OS of patients in the high-risk and low-risk groups in the TCGA cohort (P-value <.0001). * p value < 0.05, ** p value < 0.01.

To investigate the predictive value of these differentially expressed CCGs, we first carried out univariate analysis of the standardized expression of the CCGs from the LIHC datasets in the training sets to detect prognostic CCGs. In the TCGA LIHC cohort, 9 circadian genes were differentially expressed between tumor and tumor-adjacent normal tissues. Three candidate genes were significantly associated with OS, as detected by univariate Cox proportional regression analysis (**Figure 2A**). In addition, we performed LASSO Cox proportional hazard regression to screen the ultimate CCGs for the risk-score models, and found that expression of 4 CCGs correlated with OS in LIHC patients (**Figure 2B**). Therefore, we constructed a risk-score model for predicting the prognosis of LIHC patients using the calculation formula described in the Methods. We identified PER1, CRY2, CSNK1D, and FBXL21 as risk genes in the LIHC risk-score model (**Figure 2C**). Survival analysis revealed that there was a significant difference between the high-risk group and the low-risk group for OS, and being in the high-risk group significantly correlated with an poorer prognosis (LIHC: p < 0.0001) (**Figure 2D**).

### Validating the signature in the ICGC cohort

**Figure 3:**
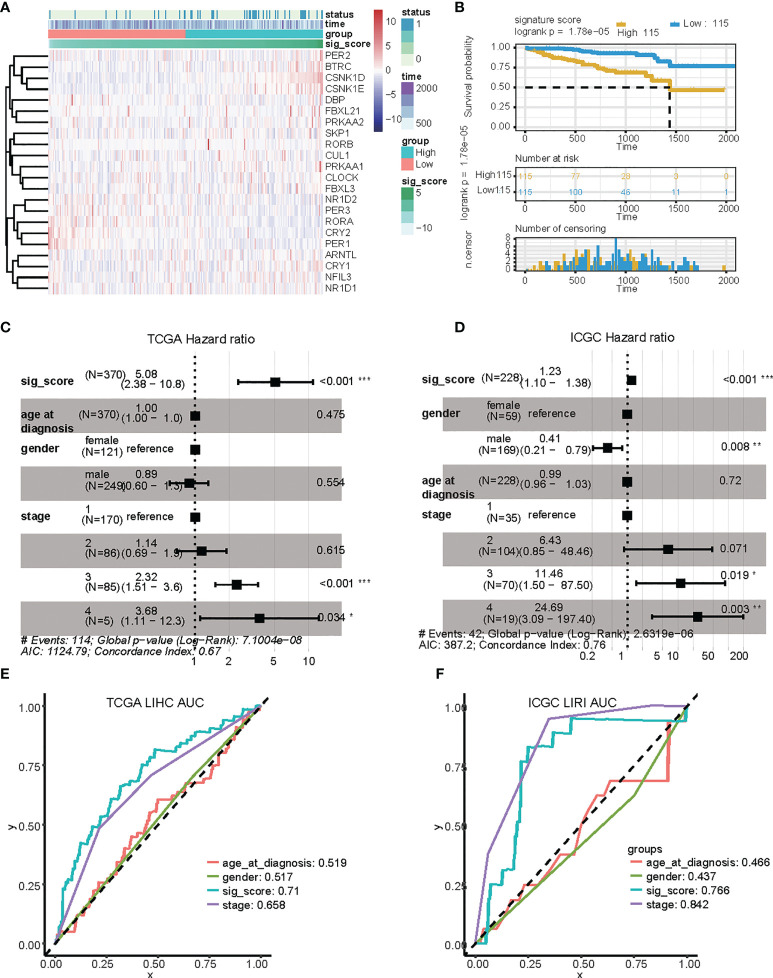
Validation of the risk score based on four signature genes from the ICGC dataset. **(A)** Distribution and expression of circadian-related genes based on the risk scores in the ICGC dataset. **(B)** Kaplan Meier curves for the OS of patients in the high-risk and low-risk groups in the ICGC dataset. **(C, D)** Multivariate Cox regression analyses of factors affecting OS in the TCGA LIHC cohort **(C)** and in the ICGC LIRI dataset **(D)**. **(E, F)** Time-dependent ROC analyses indicated that risk score had greater predictive value than other clinical features for 1-year OS in TCGA LIHC and ICGC LIRI. ROC, receiver operating curve; *, P < 0.05; **, P < 0.01; ***, P < 0.001.

We confirmed the accuracy and stability of the prognostic risk-score model using the testing sets, which included the LIRI-JP cohorts obtained from ICGC. OS was used as the indicator for comparison of the groups, and samples were split into low and high risk-score groups according to the calculated risk score. The formula was as described in the Methods. For the testing set of the LIRI type, 230 samples were separated into low and high risk-score groups. Survival analysis showed that there was a significant difference between the high and low risk-score groups (p < 0.0001, HR: 1.493, 95% CI: 1.248 to 1.787) (**Figure 3A**). To summarize, our results confirmed that the risk-score model based on CCGs signature was stable and accurate in predicting the prognosis of patients (**Figures 3B–F**).

### Identification of the circadian-based signature as an independent prognostic factor for HCC

**Figure 4:**
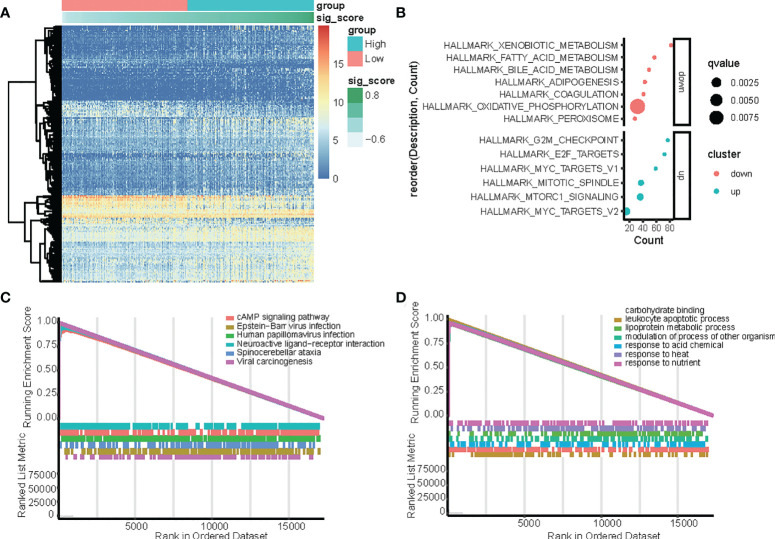
Identification of differentially expressed genes (DEGs) between the high-risk and low-risk score groups in TCGA-LIHC dataset with the cut-off criteria of |logFC|>1 and adj.P <0.05. **(A)** Heatmap of upregulated and downregulated DEGs in the TCGA-LIHC dataset. **(B)** Functional enrichment analysis of differentially expressed genes (DEGs) between the high-risk and low-risk groups. **(C, D)** GSEA analysis in the TCGA dataset according to risk score, **(C)** hallmark pathway, and **(D)** KEGG pathways.

We then investigated the accuracy and stability of the risk score for use as a clinical indicator. We analyzed seven variables, including age, gender, tumor stage, and risk-score using the TCGA LIHC and ICGC LIRI groups, with multivariate analysis and Cox proportional hazard regression, respectively (**Figures 4C, D**). Our analysis showed that risk-score was an independent prognostic indicator in both LIHC and LIRI patients. We then constructed ROC curves for the different variables to evaluate the risk-score as classifiers, and the AUC was calculated and used as the basis for evaluation (LIHC: AUC 0.71; LUSC: AUC 0.76) (**Figures 4E, F**). Our results indicated that the risk-score showed accuracy and predictability for comparing other clinical characteristics in liver cancer samples. The protein expression level of these genes in liver hepatocellular carcinoma samples was also detected *via* immunohistochemistry (IHC), as shown in **Supplementary Figure 1**.

### Functions and pathways correlated with the circadian gene-based signature

In the TCGA dataset, genes that were differentially expressed in the high-risk and low-risk groups were subjected to GSEA to evaluate the hallmark, KEGG, and GO pathways (**Figure 4**). The results indicated that tumorigenesis pathways related to G2M checkpoint, E2F target, MYC targets, mitotic spindle, and mTORC1 signal were enriched in the high-risk group, while metabolism pathways related to xenobiotic, fatty acid, bile acid, adipogenesis, coagulation, oxidative phosphorylation, and peroxisome were enriched in the low-risk group. (**Figure 4B**). In the TCGA dataset, KEGG enrichment analysis based on GSEA analysis (**Figure 4C**) suggested that CCGs modulate the cAMP signaling pathway, Epstein−Barr virus infection, human papillomavirus infection, neuroactive ligand−receptor interaction, spinocerebellar ataxia, and viral carcinogenesis. Additionally, GO analysis based on GSEA analysis (**Figure 4D**) suggested that CCGs modulate carbohydrate-binding, leukocyte apoptotic process, lipoprotein metabolic process, modulation of the process of another organism, response to acid chemical, response to heat, and response to nutrient, and the differential expression of genes between the high-risk and low-risk groups also supported that conclusion.

**Figure 5:**
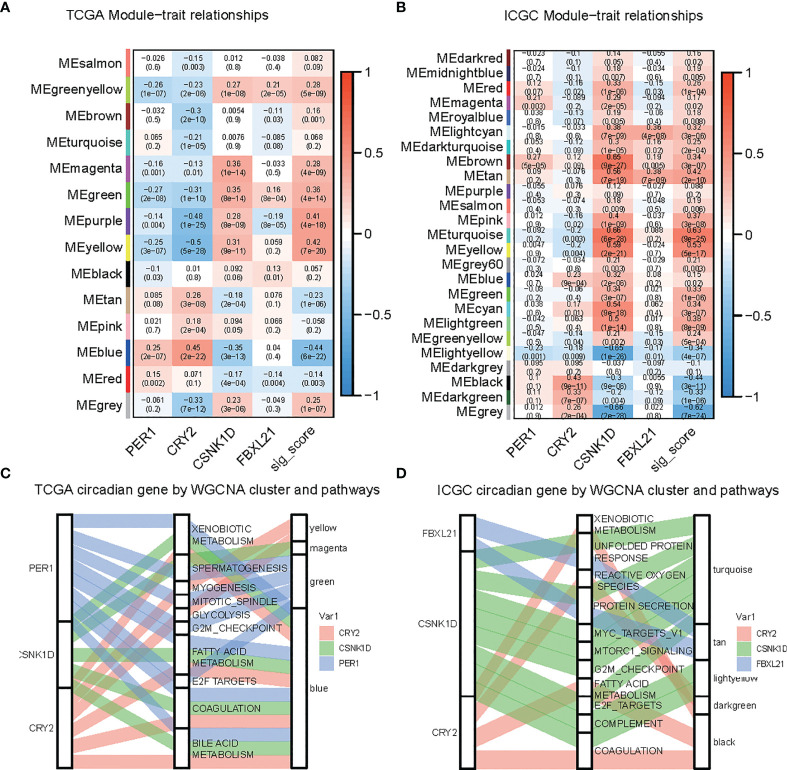
Identification of modules related to circadian genes in the TCGA-LIHC and ICGC datasets. **(A, B)** Module-trait relationships, where rows represent color modules and columns represent clinical traits (normal and tumor). Cells represent the corresponding correlation and P-value. **(C, D)** Sankey plot indicates the association between the circadian gene, hallmark pathway, and WGCNA modules.

To identify the key player in LIHC, we developed the gene coexpression network in the TCGA-LIHC and ICGC-LIRI datasets. We investigated the correlation between risk score-related CCGs and gene modules. The heatmap revealed the correlations between the modules and risk score genes in the TCGA-LIHC and ICGC-LIRI dataset (**Figures 5A, B**). Further, we found that CRY2 was negatively correlated with MYOGENESIS and GLYCOLYSIS, while others were positively correlated. Additionally, CRY2 and PER1 were negatively correlated with MITOTIC_SPINDLE and G2M_CHECKPOINT, while CSNK1D and FBXL21 were positively correlated (**Figures 5C, D**).

### Drug response and signature pathway profile based on risk score group

**Figure 6:**
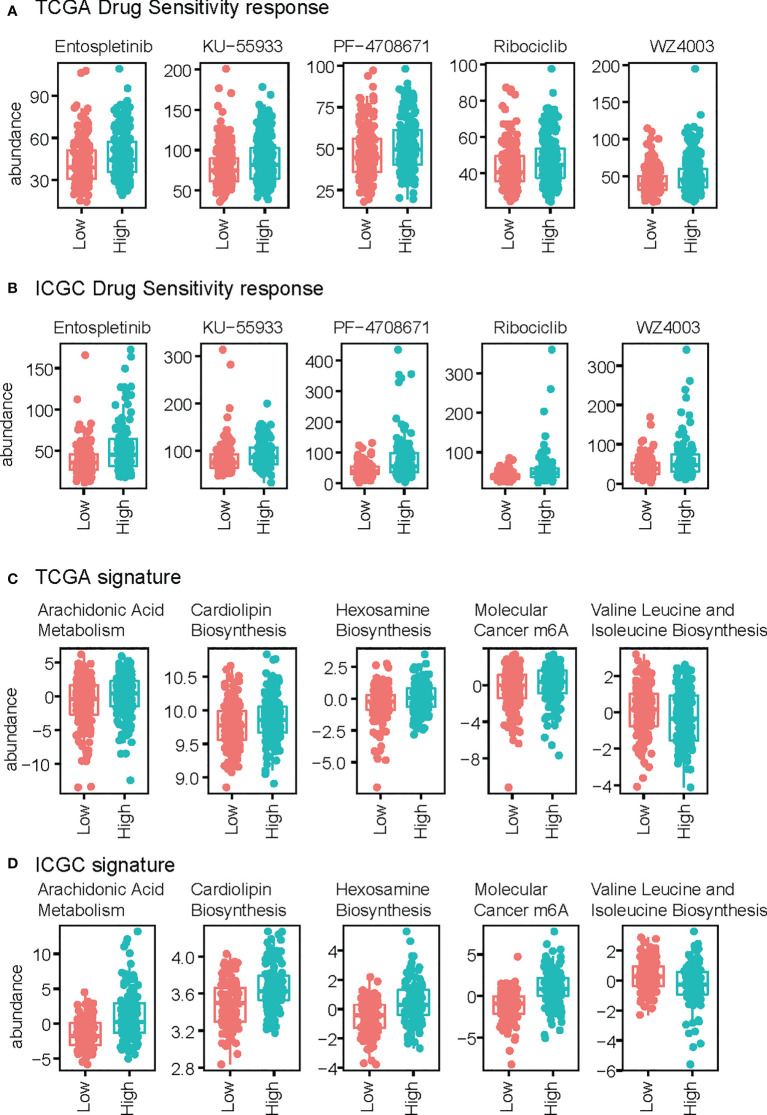
Comparison of drug response and signature score between high-risk and low-risk groups. **(A, B)** Drug response of the TCGA **(A)** and ICGC **(B)** datasets. **(C, D)** Signature scores of TCGA **(C)** and ICGC **(D)**.

We also predicted drug sensitivity according to riskScore. Entospletinib, KU−55933, PF−4708671, Ribociclib, and WZ4003 showed a positive correlation with risk score, indicating their potential role as drugs targeting liver hepatocellular carcinoma, both in TCGA-LIHC and ICGC-LIRI (**Figures 6A, B**). We next assessed the association between risk score and signature pathway in the TCGA dataset. We used the ssgsea algorithm, and found that higher risk score correlated with a higher arachidonic acid metabolism, cardiolipin biosynthesis, Hexosamine Biosynthesis, and m6A molecular cancer genesets, while lower risk score was associated with higher Valine Leucine and Isoleucine Biosynthesis (**Figure 6C**). These findings were validated in the other datasets (**Figure 6D**) supporting a difference in metabolism between the high-risk and low-risk samples.

## Discussion

Liver cancer diagnosis rates have tripled in the past three decades, and mortality has also increased (17). Thus, is it critical to identify effective biomarkers that allow creation of accurate predictive models for HCC patient OS. Ongoing investigation of metabolomics has revealed the role of metabolism in the incidence and development of cancer (18).

The circadian clock affects and drives many biological processes, and disruption to the circadian clock is implicated in breast and colorectal cancers (19, 20), with dysregulation of CCGs correlated with cancer progression (21, 22). In the current study, we established a predictive model and demonstrated that a dysregulated circadian clock correlated with hepatocellular carcinoma. The risk score model incorporating the four genes CRY2, CSNK1D, FBXL21, and PER1, based on the TCGA-LIHC dataset, was shown to be robust. These four genes have been demonstrated in previous studies to be associated with tumor development and cancer metastasis (23–26). CRY2 codes for a flavin adenine dinucleotide-binding protein that acts as a regulator of the circadian clock, and may promote growth of tumor cells (26). CSNK1D encodes an enzyme that has many roles, including in circadian rhythm, response to DNA damage, the cell cycle, functions of the cytoskeleton, and others (27, 28). FBXL21 is a gene that plays a role in circadian clock oscillation by mediating ubiquitination and stabilization of cryptochromes (29). PER1 is important in the maintenance of the circadian rhythm in cells, and its down-regulation can enhance tumor growth (25). Based on these genes, the current study showed that patients with a high-risk score had significantly reduced OS. Our predictive model was robust and accurate in correlating clinicopathological data and outcomes with higher risk scores.

We used GSEA analysis to examine the biological mechanisms driving outcomes for the two patient groups. We found that the top-upregulated HALLMARK gene sets in patients in the high-risk group were primarily associated with cell cycle-related pathways, which form the canonical signaling pathways involved in the initiation, invasion, and metastasis of tumor cells. In addition to the three common signal pathways “DNA-Repair”, “Myc-Targets-V1”, and “Myc-Targets-V2”, two other significantly enriched immune-related pathways were identified in patients in the low-risk group. The mechanistic target of the rapamycin (mTOR) signaling pathway is closely related to immune and inflammatory effects and plays an important role in activation and differentiation.

We also that predicted Entospletinib, KU−55933, PF−4708671, Ribociclib, and WZ4003 may be potential drugs for high-risk LIHC patients. Notably, a previous study supported Entospletinib monotherapy in patients with relapsed or refractory chronic lymphocytic leukemia previously treated with B-cell receptor inhibitors: the results of a phase 2 study (30) showed that Ribociclib was cytotoxic and reduced cell proliferation rate. The effect on cell viability was enhanced when Ribociclib was combined with progesterone and/or mitotane (31). Therefore, the risk score model can also be applied to predict potential drugs for treatment of LIHC. Collectively, these results indicate the accuracy and potential wide application of our model based on 4 genes for assessing the association between circadian dysregulation and cancer incidence.

There are several limitations to this study. It is a bioinformatic study without sample verification or *in vitro* and *in vivo* experiments. Moreover, the current study only focuses on gene transcription regulation. Other regulatory layers such as epigenetic, post-transcription, translation efficiency, and post-translation modification also affect the chronophysiology and pathology (32). Future investigations on experiments and prospective trials are needed to assess the predictive score of gene signature and its mechanisms.

## Conclusion

This study examined the potential role of the expression of circadian-related genes in the development of HCC. We found that 4 genes could be used to accurately predict the OS for HCC patients in the TCGA LIHC and ICGC (LIRI-JP) cohorts. Further, we identified one of the four genes, CRY2, as a possible molecular target for HCC incidence and progression. These findings provide a theoretical basis for ongoing study of circadian-related genes for predicting and treating HCC.

## Data availability statement

The original contributions presented in the study are included in the article/**Supplementary Material**. Further inquiries can be directed to the corresponding author.

## Author contributions

ZW and HH designed the study. HH, QZ, and TW performed the experiments. HH, HL, YQ, XA, XW, WY, and WG analyzed the data. ZW, HH, and CO wrote the manuscript.

## Funding

The authors would like to thank ICGC (https://www.icgc-argo.org) and TCGA (http://cancergenome.nih.gov) for data collection, cBioPortal (http://www.cbioportal.org, version v3.2.11) for the provision of data processing and customizable functions.

## Conflict of interest

The authors declare that the research was conducted in the absence of any commercial or financial relationships that could be construed as a potential conflict of interest.

## Publisher’s note

All claims expressed in this article are solely those of the authors and do not necessarily represent those of their affiliated organizations, or those of the publisher, the editors and the reviewers. Any product that may be evaluated in this article, or claim that may be made by its manufacturer, is not guaranteed or endorsed by the publisher.

## Supplementary material

Supplementary Figure 1 The Human Protein Atlas (HPA) database was used to validate the circadian genes CRY2 and CSNK1D. No data for FBXL21 or PER1 was available in the HPA database.
