# Supplementary material for: Four circadian rhythm-related genes predict incidence and prognosis in hepatocellular carcinoma
Source: Front Oncol. 2022 Nov 10;12:937403. doi: 10.3389/fonc.2022.937403 (PMC9691441; doi:10.3389/fonc.2022.937403)

CRY2
HPA037577
Male, age 65
Liver (T-56000)
Carcinoma, Hepatocellular, NOS (M-81703)
Patient id: 3196
Tumor cells
Staining: High
Intensity: Strong
Quantity: >75%
Location: Cytoplasmic/ Membranous

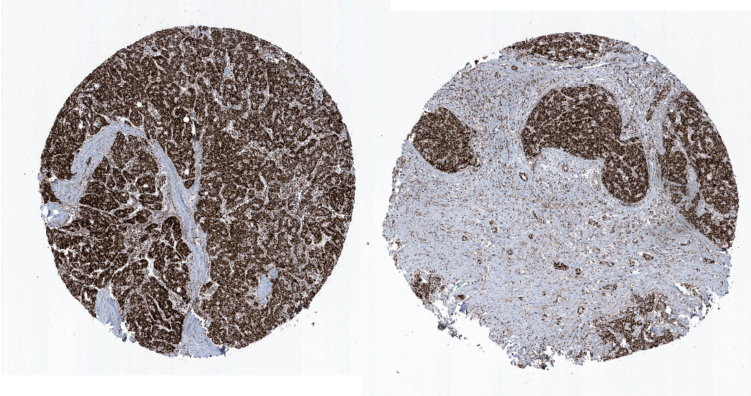

CSNK1D
CAB015410
Female, age 73
Liver (T-56000)
Carcinoma, Hepatocellular, NOS (M-81703)
Patient id: 2766
Tumor cells
Staining: High
Intensity: Strong
Quantity: >75%
Location: Cytoplasmic/ membranous/nuclear

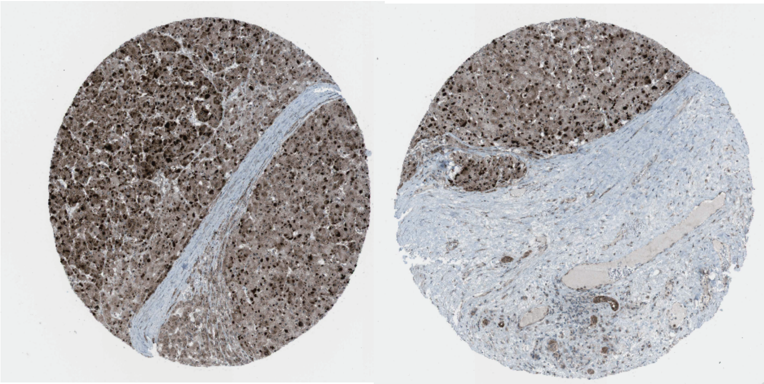

Supplement: Supplementary Figure 1 — The Human Protein Atlas (HPA) database was used to validate the circadian genes CRY2 and CSNK1D. No data for FBXL21 or PER1 was available in the HPA database. [file DataSheet_1.pdf]
